# Electronic Co-design (ECO-design) Workshop for Increasing Clinician Participation in the Design of Health Services Interventions: Participatory Design Approach

**DOI:** 10.2196/37313

**Published:** 2022-09-22

**Authors:** April Savoy, Himalaya Patel, Umber Shahid, Alexis D Offner, Hardeep Singh, Traber D Giardina, Ashley N D Meyer

**Affiliations:** 1 Purdue School of Engineering and Technology Indiana University-Purdue University Indianapolis Indianapolis, IN United States; 2 Center for Health Information and Communication (Center of Innovation 13-416), Health Services Research and Development Service Richard L Roudebush Veterans Affairs Medical Center United States Department of Veterans Affairs Indianapolis, IN United States; 3 Centers for Health Services and Aging Research Regenstrief Institute Inc Indianapolis, IN United States; 4 Center for Innovations in Quality, Effectiveness and Safety Michael E DeBakey Veterans Affairs Medical Center United States Department of Veterans Affairs Houston, TX United States; 5 Department of Medicine Baylor College of Medicine Houston, TX United States

**Keywords:** clinicians, community-based participatory research, web-based design, delivery of health care, health intervention, physicians, primary health care, videoconferencing

## Abstract

**Background:**

Participation from clinician stakeholders can improve the design and implementation of health care interventions. Participatory design methods, especially co-design methods, comprise stakeholder-led design activities that are time-consuming. Competing work demands and increasing workloads make clinicians’ commitments to typical participatory methods even harder. The COVID-19 pandemic further exacerbated barriers to clinician participation in such interventions.

**Objective:**

The aim of this study was to explore a web-based participatory design approach to conduct economical, electronic co-design (ECO-design) workshops with primary care clinicians.

**Methods:**

We adapted traditional in-person co-design workshops to web-based delivery and adapted co-design workshop series to fit within a single 1-hour session. We applied the ECO-design workshop approach to codevelop feedback interventions regarding abnormal test result follow-up in primary care. We conducted ECO-design workshops with primary care clinicians at a medical center in Southern Texas, using videoconferencing software. Each workshop focused on one of three types of feedback interventions: conversation guide, email template, and dashboard prototype. We paired electronic materials and software features to facilitate participant interactions, prototyping, and data collection. The workshop protocol included four main activities: problem identification, solution generation, prototyping, and debriefing. Two facilitators were assigned to each workshop and one researcher resolved technical problems. After the workshops, our research team met to debrief and evaluate workshops.

**Results:**

A total of 28 primary care clinicians participated in our ECO-design workshops. We completed 4 parallel workshops, each with 5-10 participants. We conducted traditional analyses and generated a clinician persona (ie, representative description) and user interface prototypes. We also formulated recommendations for future ECO-design workshop recruitment, technology, facilitation, and data collection. Overall, our adapted workshops successfully enabled primary care clinicians to participate without increasing their workload, even during a pandemic.

**Conclusions:**

ECO-design workshops are viable, economical alternatives to traditional approaches. This approach fills a need for efficient methods to involve busy clinicians in the design of health care interventions.

## Introduction

### Problem Description, Significance, and Previous Work

Participatory design describes practices for cocreating products and services with users. The co-design approach places stakeholders as peers to the intervention planners and system designers. Co-design methods have been shown to improve the development, usability, and rollout of health services interventions [1]. Co-design methods also benefit health services researchers by improving the relevance of research, showing high sustainability, and increasing subsequent collaborations [2]. Co-design has a long history of health services applications. Recent examples with health care staff include improving hospital palliative care [3], primary care decision support for antibiotics prescribing [4], and primary care artificial intelligence–based documentation assistants [5].

Traditionally, co-design workshops are conducted in person [6]. This enables prototyping with low-tech or rudimentary materials to maximize accessibility and engagement. However, traditional co-design is difficult. Barriers include transportation, retention, and for staff, reluctance in using personal time to attend workshops [7]. The COVID-19 pandemic made some of these barriers more severe after organizations activated safety protocols that effectively ended colocated meetings. The COVID-19 pandemic also increased the use of online meetings, supporting a potential adaptation of the co-design workshop format for distributed work groups.

Online meetings have increased participation from hard-to-reach patient stakeholder populations [8] and demonstrate potential for reaching clinicians whose experiences may be underrepresented [9]. Web-based co-design provides flexibility in location, which addresses the barrier of transportation of people to a central physical location. Time commitment remains a major barrier to recruitment and retention; clinicians were busy even before the present pandemic. An abbreviated method for co-design, however, may overcome such time-related barriers.

### Objective

There is a pressing need to adapt traditional methods of co-design workshops, including delivery, facilitation, materials, activities, and duration, to increase clinician participation in the design of health care interventions [8]. Our objective was to adapt co-design workshops to encourage primary care clinicians’ participation with special consideration of their increased workload and safety during the pandemic.

### Application to a Health Services Problem: Case Study

Clinicians in ambulatory care services, including primary care, usually order diagnostic tests for their patients to investigate patient symptoms. Clinicians are expected to see the results, interpret them, plan a treatment if needed, and communicate results to the patient (even if test results are not available until after the patient has left the health care facility). Failure to follow up on abnormal test results leads to delayed and missed diagnoses. These failures happen in approximately 7% of abnormal lab results and 8% of abnormal imaging results [10,11]. As indicated in the National Academies of Sciences, Engineering, and Medicine’s Report—Improving Diagnosis in Health Care—diagnostic testing is a key part of the information gathering process within the diagnostic process [12]. Failure to follow up on key pieces of information gathered in this process (ie, abnormal test results) can lead to diagnosis and treatment delays [13,14]. To combat this problem, the health care system operated by the United States Department of Veterans Affairs (VA) requires that test results requiring no action to be communicated to patients within 14 days after availability and results requiring an action to be communicated within 7 days (based on Veterans Health Administration Directive 1088).

Missed follow-up of test results occurs due to several sociotechnical factors [15]; interventions to deliver feedback to improve test result follow-up also need to be sociotechnical [16]. We used participatory methods involving clinicians, including co-design workshops, to identify possible interventions to deliver feedback that could improve follow-up of test results in VA primary care.

## Methods

### ECO-design Workshop

**Figure 1:**
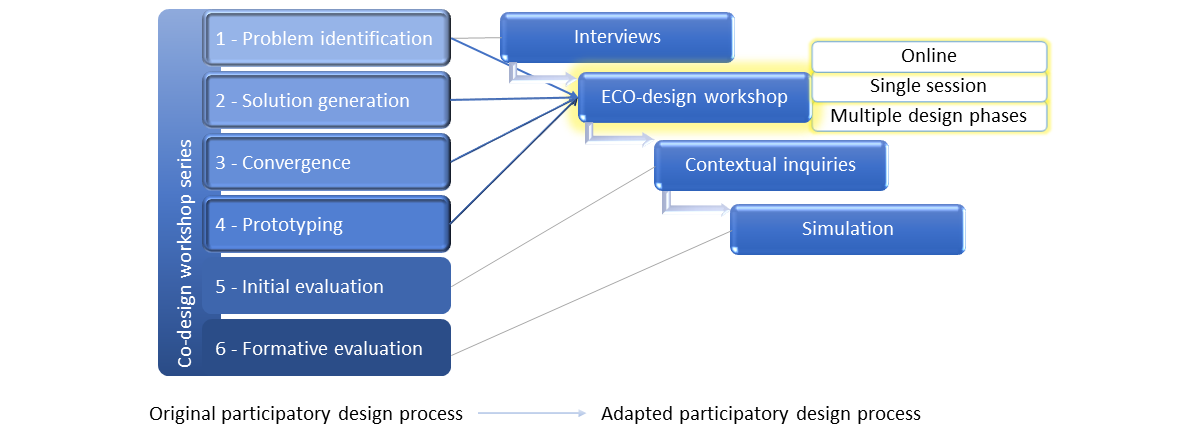
Structure of an adapted co-design (ECO-design) process.

Our adaptations were intended to create an economical and electronic (ECO) version of traditional co-design workshops, allowing remote (ie, in their own ecosystem, minimizing the need for travel) and efficient (ie, brief, minimizing time burden) physician participation. Our ECO-design workshop can also be conceptualized as an eco-mode for traditional co-design workshops. We modeled our approach on Reddy et al’s approach [2], which included clinician stakeholders (pharmacists) in the design of a health services intervention. Reddy et al’s approach [2] included detailed descriptions of a co-design process with the following 6 steps: “(1) problem identification, (2) solution generation, (3) convergence, (4) prototyping, (5) initial evaluation, and (6) formative evaluation.” We adapted this 6-step process (Figure 1) for distributed groups with limited availability for participation. Our process adaptations were driven by the COVID-19 pandemic’s constraints on primary care clinicians. The most impacted aspect of Reddy et al’s published design process [2] was the in-person co-design workshop. Their process involves 6 co-design workshops to be attended by all participants. Each workshop lasts about 2 hours, and workshops are expected to be scheduled 4-6 weeks apart to permit analysis of completed sessions and planning of future sessions [2]. During the pandemic, clinicians were less available to participate in any type of research activity that lasted more than an hour, nonessential travel was prohibited for clinicians and researchers, and there was a limit on the number of people in any given room, consistent with social distancing policies. Therefore, our adaptations to the co-design workshop were needed to enable a large group of clinicians to participate in workshops lasting 1 hour or less.

### Recruitment: Setting, Sampling, and Ethics

We used convenience sampling with an emphasis on homogeneity [17]. Participants were recruited from the primary care clinics of a large medical center in Southern Texas. Any clinician in this department was considered a possible participant. Recruitment was done via email, with assistance from a senior primary care clinician at the medical center. We offered each potential participant a medical textbook as an incentive. We were permitted to use a recurring meeting time, which was normally reserved for a department-wide journal club, for the co-design workshops. Because potential participants were already scheduled to attend this meeting of the journal club, we avoided adding to their daily loads. Participants indicated their consent to recording via spoken response to the facilitator.

### Ethics Approval

This study received expedited approval (protocol H-44363) by the Institutional Review Board at Baylor College of Medicine and the Research and Development Committee at Michael E DeBakey VA Medical Center.

### Logistics: Data Collection Methods, Instruments, and Technologies

#### Technology

The workshops were organized using the medical center’s primary videoconferencing software—Microsoft Teams (Microsoft Corp). Microsoft Teams provided scheduling reminders (through the medical center’s calendar software, Microsoft Outlook), included automatic log-in for participants on work-furnished devices, offered a method for sharing files via the chat space, and offered a method for recording audio and video from the workshops. Closed captioning and autotranscription were not available with this software at the time of the workshop. Participants were able to mute and unmute their cameras and microphones. Names of signed-in participants were displayed onscreen; phone numbers were shown for those who dialed in to the telephone bridge.

#### Materials

We created an electronic workbook for the ECO-design workshops. The workbooks were emailed to participants before the workshops and posted in the chat during the session. The contents of the workbook (Multimedia Appendix 1, part A) were organized to align with the workshop agendas and contained writing prompts for each intervention type to support group activities. The workbooks were designed to capture participants’ individual thoughts before engaging in group discussions. With the workbooks, participants were able to elaborate on ideas independently before, during, and after discussion. The chat function provided a secondary method for collecting participant contributions.

#### Virtual Rooms

To minimize participants’ time burden, the workshops took place during a regularly scheduled meeting time. We started the ECO-design workshops in one virtual room, where the topic and relevant background information related to the problem we were trying to solve was introduced (ie, failure to follow up on abnormal test results). When the workshops were conducted, Microsoft Teams’ breakout room feature was new and potentially unfamiliar to some users and unavailable to others, so we emulated breakout rooms by creating 4 additional Microsoft Teams meetings and inviting participants to leave the main room and enter their assigned breakout room.

Participants in each room were asked to work on one of 3 feedback interventions we developed from the literature in the problem domain [18-22]. For purposes of our project, we labeled each of the 3 intervention types as follows: “social” (ie, a conversation guide for one’s supervisor), “technical” (ie, an electronic data dashboard), and “sociotechnical” (ie, an email message or template). The separate rooms enabled focused exploration of all the intervention types simultaneously and encouraged participation within smaller groups [8].

#### Research Team and Roles for the ECO-design Workshops

Workshops were facilitated by a multidisciplinary research team, whose members all held advanced degrees in relevant fields: DrPH in management, policy and community health (US), PhD in cognitive psychology (ANDM), PhD in social work (TDG), PhD in industrial engineering with emphasis on human-computer interaction (AS), PhD in informatics (HP), and MPH in health promotion and health education (ADO). There were 9 team members in total, 2 per breakout room (eg, 1 facilitator and 1 notetaker) and 1 person assisting participants in the main room.

#### Communication

A group text message via team members’ cellular phones was critical to the success of the workshop because the technology did not support communication across breakout rooms. Text messaging was used to ensure the leads of each room completed all segments of their workshops in the allotted time.

**Figure 2:**
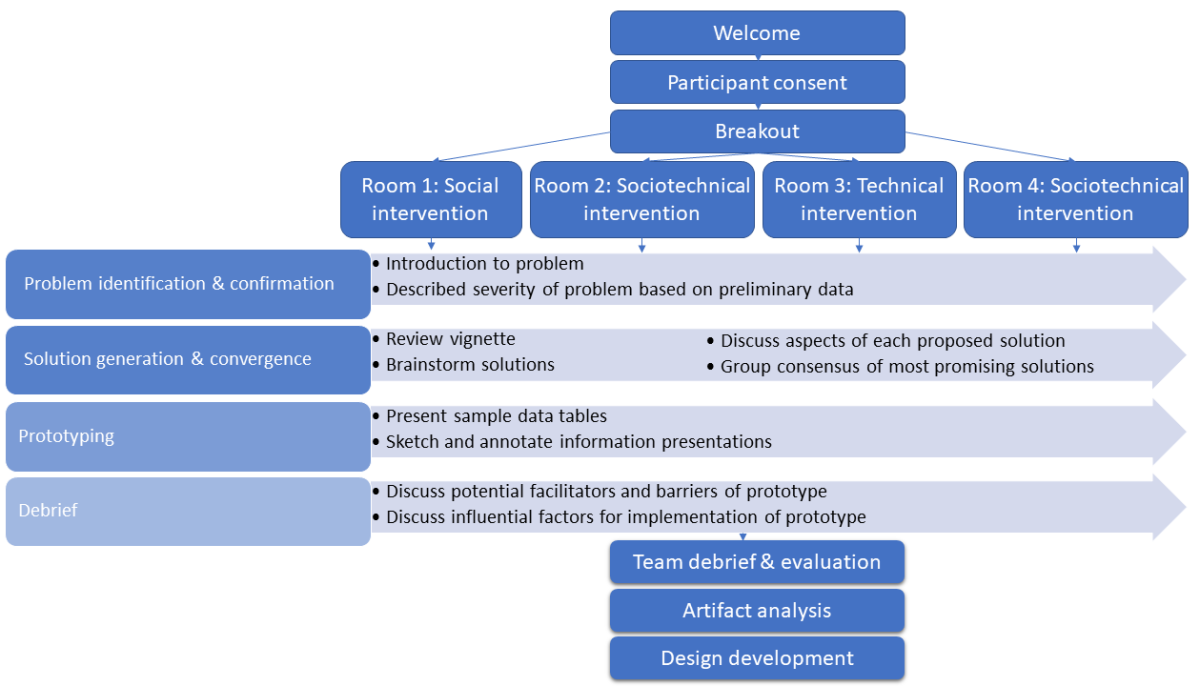
ECO-design procedure.

Workshop segments (Figure 2) were timed as follows: welcome and consent (3 minutes), problem identification and confirmation (4 minutes), solution generation and convergence (13 minutes), prototyping (35 minutes), and debrief (5 minutes). During the workshops, facilitators read from an annotated slide deck (Multimedia Appendix 1, part B). Participants were encouraged to turn on their cameras, but it was not required. First, one facilitator (AS) introduced the research team members, described the aims of the study, and the objective of the ECO-design workshops. Second, spoken consent was sought for recording audio and video from the main and breakout sessions. Third, from the list of attendees, we assigned participants to four workshops by surname. After posting hyperlinks to four breakout rooms in the chat window of Microsoft Teams, we instructed participants to enter the breakout session assigned to their surname. Each breakout session was assigned either the social, technical, or sociotechnical intervention. Each breakout session was facilitated by a coauthor with experience in research interviewing (AS, TDG, US, and HP). A second facilitator in each session began audio and screen recording, took notes, and monitored chat content. Both facilitators shared their screens at various points in the session to display notes and enable viewing, annotating, and manipulation of the prototype. Another team member stayed in the main room to help participants with technical issues and to prompt team members in each room regarding when to move on to the next topic or end the session (ANDM).

In each virtual room, the session started with the following prompt:

Consider this: your supervisor walks into your office and says, “a number of your patients have abnormal test results with no documented follow-up. You will need to address these delays.” What is your initial reaction?

Participants were asked to respond to this prompt in their workbooks. Next, participants were asked to design the subsequent interaction using an assigned mode (Figure 2; Table 1). One room was assigned to a dashboard visualization (facilitated by AS), another to a conversation guide (facilitated by TDG), and the remaining two to email template (facilitated by US and HP). The workbooks supported participation throughout the phases, steps, and tasks in the ECO-design workshops.

**Table 1:** ECO-design workshop tasks and complementary workbook prompts.

Workshop tasks		Workbook prompts
**Problem identification and confirmation**		
		What is your initial reaction? What would be your response? What would be the first thing you do after your supervisor left the room?
**Solution generation and convergence**		
	Conversation guide	How would you improve the conversation or supervisor dialogue? How would you improve the conversation started in the scenario?^a^ Discuss tone, language, and communication mode.^a^ Who should be the one initiating the conversation?^a^ What information would best support the conversation?^a^
	Email template	What should be the subject? What needs to be communicated in this text? When should this email be sent? Should anyone be CC’d? What should be the subject?^a^ What needs to be communicated in this text?^a^ Tone Links Attachments When should this email be sent?^a^ Messaging frequency.^a^
	Dashboard prototype	If a dashboard existed, where would you expect to find it (ie, necessary navigation)? How would you like to be notified of updates or new information? Where would you like to see this dashboard?^a^ How would you expect to navigate to it?^a^
**Prototyping**		
		How would this help you understand and address the problem? How would you redesign this table? How would this help you understand and address the problem?^a^ What would be your first step or question after seeing these data?^a^ How would you redesign this table?^a^ Add Delete Rearrange What would be the most helpful time frame?^a^
**Debriefing**		
		How does this compare to existing performance data that are available to you? How would you like to gain access to this type of data?^a^

^a^Optional prompts.

Then, while still within the breakout sessions, participants were asked to review the content of tables corresponding to a summary data presentation across many patients and data presentations for individual patients. We prepared these tables before the workshop. In both sections, participants were encouraged to add, remove, or reorganize the data to aid in understanding and facilitate appropriate action (Multimedia Appendix 1, part C).

At the end of the workshop, participants were asked to send their completed workbooks either via Microsoft Teams to the breakout room facilitator or via email to the study coordinator. Finally, after being asked to share their completed workbooks with their facilitators, participants were dismissed. Recordings of the main workshop and breakout sessions were stopped here and processed using Microsoft Teams. Workbooks and recordings were stored securely on an access-controlled network file server.

### Workshop Evaluation and Analysis

A team debriefing session occurred 2 days after the ECO-design workshop. The meeting was held via Microsoft Teams for 1 hour. The agenda items included ideas for improvement regarding logistics (eg, what went well, how engaged the participants were, some weaknesses of the methods, and where we can refine the methods), major themes and ideas, debriefing details according to breakout room, initial thoughts about the data, and next steps for the team. Notes were taken during the meeting and shared with the research team.

## Results

### Participants

We were provided with the names of 49 people (of those, 1 was later identified as a nonclinician and was excluded). A total of 28 clinicians responded to our invitation, and all 28 attended the workshop. We assigned 8 people to the dashboard intervention workshop, 5 to the conversation guide intervention workshop (including the nonclinician), and 6 and 10 to the first and second email workshops, respectively. The workshops were conducted in January 2021.

### Outcomes

**Figure 3:**
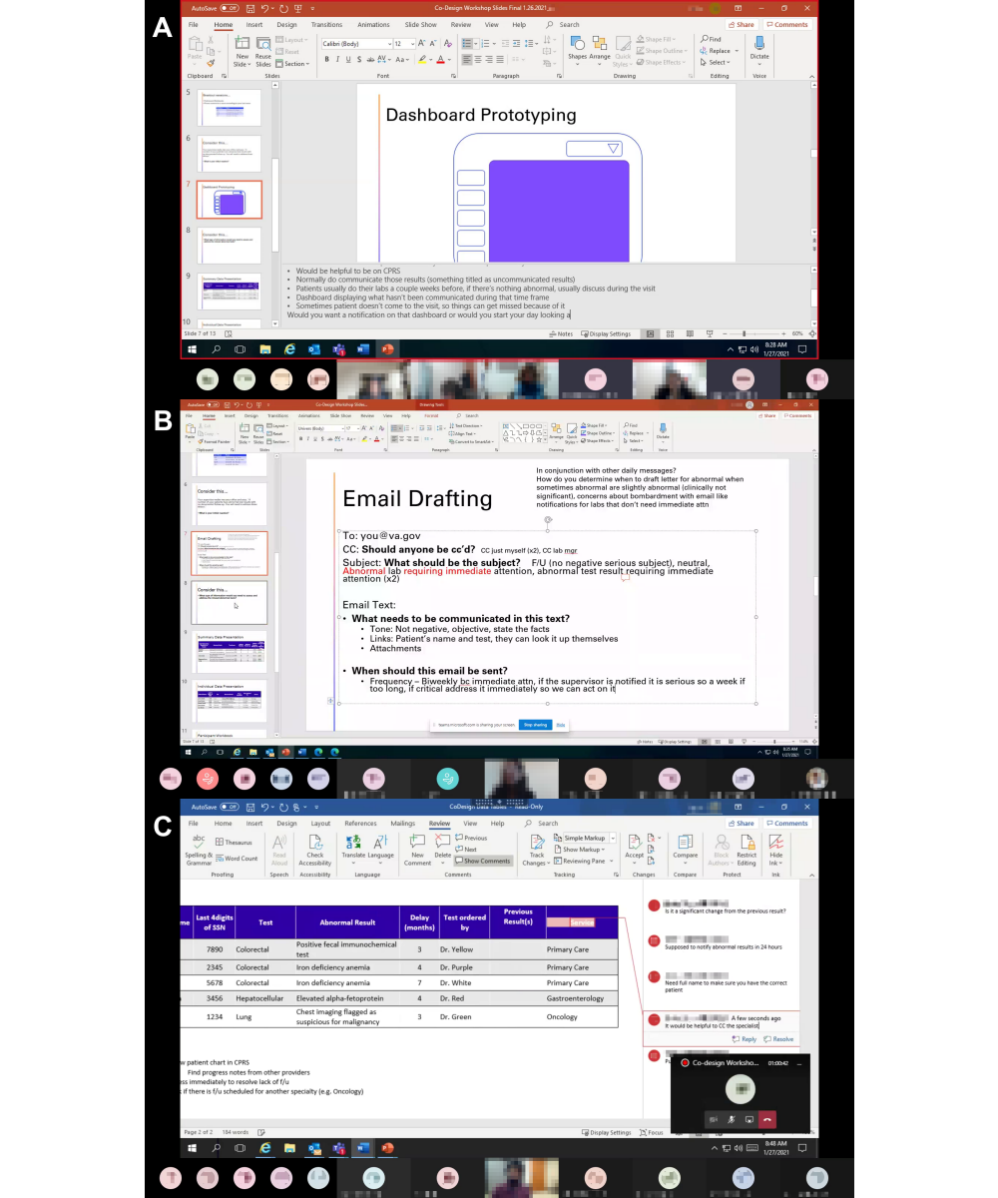
Part A: ECO-design workshop participants lay out a technical intervention (data dashboard) to improve test-result management. Part B: workshop participants co-create a sociotechnical intervention (email message) to improve test-result management. Part C: workshop participants mark up a hypothetical table of unresolved results.

Figure 3 shows examples of ECO-design from the parallel workshops. Participants in the technical intervention workshop laid out a data dashboard (Figure 3A), while participants in one of the sociotechnical intervention workshops proposed the content of a supervisor’s email message (Figure 3B). In the other sociotechnical intervention workshop, participants marked up tables indirectly through the second facilitator (Figure 3C).

The ECO-design workshops provided data and artifacts similar to traditional co-design workshops. We were able to perform traditional analysis and next steps that led to the development of an empathy map (ie, a graphical description of clinician stakeholders). The workshops also informed user interface prototypes with varying fidelity levels. The sociotechnical intervention and social intervention workshops contributed to high-fidelity prototypes of email and script templates. The technical intervention workshop attributed to a medium-fidelity prototype of a dashboard.

### Debriefing and Evaluation

We held a debriefing meeting after the ECO-design workshop. In the meeting, we discussed ideas for improvement in logistics, such as assigning a dedicated person to Microsoft Teams troubleshooting, ensuring participants used the organization’s current version of Microsoft Teams, helping participants join their assigned breakout room in a timely manner, and adding expedited introductions at the beginning of the workshop. Most participants did not use their cameras during the workshop, which we agreed should be addressed for the next workshop. However, no specific solution was reached during the debriefing meeting. Some participants were more active than others, whether speaking aloud or contributing to the chat or both. Finally, only 5 participants returned workbooks from the ECO-design workshops, with an average of 3.6 questions answered completely; therefore, finding a way to ensure completion and timely receipt of the workbooks is needed.

We identified several challenges related to facilitation. In traditional workshops, a brief introduction of all participants, optionally with an icebreaker activity, can help participants build on each other’s strengths. We omitted this step with regard to our time limit. However, most clinicians knew each other as coworkers. Moderating the sessions in a way where we ask individual participants about their thoughts and suggestions instead of waiting for the respondents to jump in and reply may encourage broader participation. We could not see whether participants were filling out the workbook as we were moving forward with the slides; future workshops should periodically remind participants to fill out their workbooks throughout the workshop. For the future, we plan to incorporate tools like digital whiteboards or live polling to verify or encourage individual and group participation.

### Recommendations for Future ECO-design Workshops

Table 2 summarizes our observations and corresponding recommendations.

**Table 2:** Recommendations for implementing ECO-design workshops.

Workshop aspect or dimension	Observation or lesson (facilitator or barrier)	Recommendation
Recruitment and scheduling	Breakout rooms allowed exploring ideas in parallel (facilitator). No travel required (facilitator). Reduced time burden on clinicians (facilitator). No peer introductions or icebreaker activities (barrier); acceptable if participants already know each other. Invitees via digital recruitment may not be from the desired stakeholder group, owing to outdated or missing information (barrier).	Take advantage of existing meetings; this will help with recruitment opportunities because adding another appointment to primary care clinicians’ schedules will increase their workload. We advise group sizes of 5 for a small group to 10 for a large group. Have a plan for excusing excluded participants or removing their data afterward.
Technology or videoconferencing software	Flexible teleconferencing means some people can dial in (facilitator). Too many dial-in users or users with no camera or disabled cameras make it difficult to read the room, that is, assess nonverbal communication (barrier).	Use videoconferencing software with a breakout room feature. Explicitly encourage participation via video if participants are able to do so. Collaborative editing tools (eg, a digital whiteboard) should be integrated in the videoconferencing software. Games using these tools can foster interactive creation of solutions (eg, semantic environment [23]). Audio and video recording sessions; ensure software allows recording of breakout rooms in addition to the primary room.
Facilitation	Participants prompted or self-identified as experts or as having more experience to weigh in more at different points of the workshop (facilitator).	Assign at least three people to facilitate the session (moderator, notetaker, and technical facilitator).
Time and activities	Established relationship among participants that allowed us to save time on introductions (facilitator).	Allocate time in the co-design workshop to describe and confirm the problem only (the problem identification portion has potential to run long. Use other methods to define the problem before the co-design workshop). Participant and team debriefs are essential. Allow time for participants to evaluate the session (present participants with a short web-based survey or at least a rating scale with a section for free-text comments.
Data collection	More difficult to engage participants in completing workbooks or other tasks in online meetings (barrier).	Electronic workbooks can be used as a data collection tool. Collect workbooks before the end of the session whether they are finished or not. Most likely, participants will not have time to edit before or after the session. If they leave with the file, do not expect it to be sent later. Save the chat text, as it is a great source for data analysis.

## Discussion

### Summary

This is one of the first published studies to explore a web-based participatory design with primary care clinicians. Having an effective approach to conducting design workshops in primary care is important in addressing ongoing primary care concerns and priorities, from care coordination delays to clinician burnout [24]. When the COVID-19 pandemic limited clinicians’ participation in participatory design, we adapted traditional in-person co-design workshops to web-based delivery and adapted co-design workshop series to fit within a single 1-hour session. Implementing the ECO-design workshop approach, clinicians codeveloped 3 different types of feedback interventions. Our results demonstrate the feasibility of ECO-design workshops and describe fundamental considerations for future ECO-design workshops with clinicians.

### Lessons Learned

Similar to web-based participatory design series conducted in other contexts or domains (eg, [8,25]), we found advantages, disadvantages, and opportunities for improvement related to recruitment, software, and facilitation. Some of our findings were tied to specific workshops or interventions, while other findings were more universally applicable. In addition, working with clinicians invoked considerations that are unique to the health care context.

The ECO-design format may attract more participants. Recruitment may have been aided by the ease of accepting invitations to online meetings. Due to policies at the health care institution, the incentives offered to clinicians were limited and not enticing, even for the condensed online workshop. We could offer books but not money or food. Alternatives should be considered. During the workshops, we noticed varying impacts of technology, intervention, and clinician characteristics on participation.

The participants’ ability to maneuver, edit, or manifest prototype ideas easily is essential. Our web-based platform was more challenging for clinicians than expected and was a major trade-off that needs to be considered for future ECO-design workshops. Adding collaborative software to the workshop would have provided more opportunities for participants to manipulate the prototypes. However, there were potential disadvantages, including additional costs, learning curve or familiarity with new software, and increased window switching among software programs. In the health care context, additional safety considerations include information security and related approvals for software due to privacy concerns.

Among the workshops and intervention types, participation and facilitation differed. In the technical (ie, dashboard) and sociotechnical (ie, email template) intervention workshops, we witnessed the most active participation. Despite limitations with the software, it appeared that the direct manipulation of onscreen elements encouraged participation. In contrast, we observed lower participation or engagement in the social (ie, conversation guide) intervention workshop, which may have been the most emotionally evocative. Clinicians discussed similarities to other difficult professional conversations. We were unable to determine the location or physical environments (eg, home office, team workroom, or clinic) of participants. Even if the meetings are online, it is important that participants are in safe, trusted physical and virtual environments to better support these types of activities.

Lastly, we found that a group of 10 participants was too large for small group discussions and activities, especially for a 1-hour session. There was not enough time to give everyone a chance to comment (albeit some participants preferred not to comment). Given the ease of being able to log in and only observe, engagement levels in online meetings might be decreased compared to traditional formats across contexts. Clinician participation varied as expected. Although some clinicians spoke dominantly, others used the chat functionality to contribute to the workshop. Established relationships and knowledge of expertise among clinicians aided facilitation, as participants explicitly asked particular colleagues to speak up on certain topics.

Overall, ECO-design workshops demonstrated an economical alternative to traditional co-design workshops. Specifically, we were able to minimize physician time burdens and travel costs for both participants and researchers. To maximize the level of engagement and manipulation of low-tech or physical tools, traditional co-design workshops may still be preferred when participants are colocated and technology is limited. Nonetheless, ECO-design workshops could serve as a complementary approach to traditional co-design workshops. Therefore, we recommend ECO-design or hybrid co-design workshops for the added benefit and cost-effectiveness (including time cost) of participants from multiple sites and institutions.

### Limitations and Future Work

All participants were from a single site of a single health care institution and were members of the same journal club. Although the use of the journal club enabled the workshops to occur without adding additional meetings to primary care clinicians’ schedules, this potentially introduced selection bias. For the next phase of our participatory design process, we will include primary care clinicians from other sites. Moreover, some people participated more than others in our workshops; this, however, is likely to happen in traditional co-design workshops as well and should be addressed similarly (ie, by asking questions of the quieter individuals throughout the meeting). Future workshops may include gamification to increase participation. It can be hard to motivate participation from everyone, and some may not be attending fully to the workshop because of multitasking.

We tested only one videoconferencing software platform. Other software that may have been helpful in group or collaborative editing (eg, a digital whiteboard) was not yet available in the organization or would have incurred additional costs. Future studies could conduct ECO-design workshops with other videoconferencing software and compare outcomes and experiences.

During the COVID-19 pandemic, certain aspects of traditional research design were not feasible. Pandemic restrictions and increased clinician workload prohibited additional data collection or evaluation (eg, comparative evaluation with a traditional workshop or postworkshop participant surveys). Future studies should implement the aforementioned aspects to inform the next iteration of ECO-design workshops.

### Conclusions

The ECO-design workshop enabled primary care clinicians to participate in the design process of multiple types of interventions for obtaining feedback about test result management. Our adaptations provided data and artifacts that supported a participatory design process within the amplified time constraints of primary care clinicians and safety protocols imposed by the COVID-19 pandemic. From our adapted co-design workshop, we were able to develop a prototype for each intervention type. Therefore, the ECO-design workshop is a feasible alternative to traditional in-person co-design workshops.

## Appendix

Multimedia Appendix 1 Workbook, workshop script, and data tables for discussion.

## Abbreviations

HSR&D: Health Services Research and Development
VA: Department of Veterans Affairs
VHA: Veterans Health Administration
