# Supplementary material for: Electronic Co-design (ECO-design) Workshop for Increasing Clinician Participation in the Design of Health Services Interventions: Participatory Design Approach
Source: JMIR Hum Factors. 2022 Sep 22;9(3):e37313. doi: 10.2196/37313 (PMC9539640; doi:10.2196/37313)
Supplement: Multimedia Appendix 1 [file humanfactors_v9i3e37313_app1.docx]

# Online Supplement

## Part A: Workbook

**Codesign Workshop**

**to Improve Test Result Follow-Up**

**Participant Workbook**

**Room #____**

1. **Consider this...**

What is your initial reaction?

1. **Conversation drafting (If discussed in your group)**

How would you improve the conversation or supervisor dialogue?

Revisions:

*Hello,*

1. **Email drafting (If discussed in your group)**

Should anyone be cc’d?

What should be the subject?

What needs to be communicated in this text?

When should this email be sent?

1. **Dashboard crafting (If discussed in your group)**

If dashboard existed, where would you expect to find it [necessary navigation]?

How would you like to be notified of updates or new information?

1. **Consider this…**

What type of information would you need to assess and address the missed abnormal tests?

1. **Summary data presentation**

How would this help you understand and address the problem?

How would you redesign this table?

1. **Individual data presentation**

How would this help you understand and address the problem?

How would you redesign this table?

1. **Do you have additional comments?**

**At the end of the session- please upload a copy of this to the chat or email to:**

**Thank you!**

## Part B: Workshop script

Hello, thank you for agreeing to take part in this co-design workshop for the study [Study_Name]

Slide 1 - My name is [Introduce research team members…]. We will be facilitating this workshop and taking notes.

Slide 2 – This is the agenda for today. Read agenda.

Slide 3 - The purpose of this study is to **[INSERT TEXT OR READ FROM SLIDE]**. Our goal is to **[INSERT TEXT OR READ FROM SLIDE]**.

Slide 4 – You may be wondering, “What is a CoDesign Workshop?” It is **[INSERT TEXT OR READ FROM SLIDE].** In this workshop, you will be asked to **[INSERT TEXT OR READ FROM SLIDE].** Your participation is completely voluntary and will not affect your employment in any way. You may skip any question or stop the interview at any time, for any reason, without consequence. This workshop will be recorded and then encrypted to maintain confidentiality.

- Any questions?
- Do you consent? Everyone say Aye.

Instruct everyone to break out to rooms. Describe how everyone is assigned to different rooms.

**[Begin recording.]**

This is [Study_Name] CoDesign Workshop, Room [say Last name] and today’s date is [say date].

Let’s get started…

Slide 5 - **[INSERT TEXT OR READ FROM SLIDE]**

Slide 6-

Slide 7-

Slide 8-

Slide 9-

Slide 10- **[INSERT TEXT OR READ FROM SLIDE]**

Proposed rooms (Facilitator, Note-taker, Topic):

1 (Author_1, RA_2, Dashboard),

2 (Author_6, RA_1, Convo),

3 (Author_3, Author_4, Email),

4 (Author_2, RA_3, Email)

## Part C: Data tables for discussion

Table 2. Fictitious panel-level (summary) data shown to all participants

| Test Ordered for Suspicion of Cancer | Abnormal Result | Timeframe | Unique Patients | Delayed Follow-up | Unique Patients Delays (%) | Delays Compared to National Data |
| --- | --- | --- | --- | --- | --- | --- |
| Bladder | High grade hematuria | January-June 2020 | 49 | 4 | 8.2% | ABOVE |
| Breast | Abnormal mammogram | January-June 2020 | 9 | 2 | 22.2% | BELOW |
| Colorectal | Iron deficiency anemia or positive fecal immunochemical tests | January-June 2020 | 84 | 27 | 32.1% | SAME |
| Hepatocellular | Elevated alpha-fetoprotein | January-June 2020 | 5 | 1 | 20.0% | SAME |
| Lung | Suspicious for malignancy | January-June 2020 | 46 | 16 | 34.8% | BELOW |

Table 3. Hypothetical patient-level (individual) data shown to all participants

| Patient Name | Last 4 digits of SSN | Test | Abnormal Result | Delay (months) | Test ordered by | Service |
| --- | --- | --- | --- | --- | --- | --- |
| John Smith | 1234 | Lung | Chest imaging flagged as suspicious for malignancy | 3 | Dr. Green | Oncology |
| Jane Smith | 5678 | Colorectal | Iron deficiency anemia | 7 | Dr. White | Primary Care |
| Jonah Smith | 3456 | Hepatocellular | Elevated alpha-fetoprotein | 4 | Dr. Red | Gastroenterology |
| Joan Smith | 7890 | Colorectal | Positive fecal immunochemical test | 3 | Dr. Yellow | Primary Care |
| Joe Smith | 2345 | Colorectal | Iron deficiency anemia | 4 | Dr. Purple | Primary Care |
